# LC–MS/MS and GC–MS based phytochemical perspectives and antimicrobial effects of endophytic fungus *Chaetomium ovatoascomatis* isolated from *Euphorbia milii*

**DOI:** 10.1007/s00203-022-03262-5

**Published:** 2022-10-04

**Authors:** Kamel H. Shaker, Moustafa M. Zohair, Amal Z. Hassan, Heba-tollah M. Sweelam, Warda E. Ashour

**Affiliations:** 1 Chemistry of Natural Compounds Department, Pharmaceutical and Drug Industries Institute, National Research Center, El-Behoos st, Dokki, Giza, 12622 Egypt; 2 Chemistry of Natural and Microbial Products, Pharmaceutical and Drug Industries Institute, National Research Center, El-Behoos st, Dokki, Giza, 12622 Egypt; 3 Graduate School of Bioresource and Bioenvironmental Sciences, Kyushu University, Fukuoka, 819-0395 Japan

**Keywords:** Fungal endophytes, Antimicrobial, *Euphorbia milii*, LC–MS/MS, GC–MS, *Chaetomium* sp. metabolites

## Abstract

The antimicrobial activity of endophytic fungi isolated from *Euphorbia milii* was evaluated against Gram-positive, Gram-negative bacteria, unicellular yeast, and filamentous fungi. *Chaetomium ovatoascomatis NRC* was identified morphologically and genetically as the most active strain. The total ethyl acetate extract of *C. ovatoascomatis NRC* demonstrated significant antimicrobial activity against Gram-negative; *Escherichia coli*, *Salmonella enteric*, and fungi;* Aspergillus niger* with MIC of 62.5 ug/ml. Whereas *n*-hexane fraction demonstrated broader activity against Gram-positive; *Bacillus subtilis*, *Lactobacillus cereus*, Gram-negative; *Escherichia coli* and *Salmonella enteric*, fungi; *Candida albicans* and *F. solani*. LC–MS/MS analysis of ethyl acetate strain extract and GC–MS analysis of the *n*-hexane fraction were used to identify the metabolites of the strain extract. LC–MS/MS determined three major metabolites with potential antimicrobial activities including grevilline B, aflatoxin G2 and apigenin. GC–MS analysis of *n*-hexane fraction tentatively identified 30 compounds, where 9,12-octadecadienoic acid methyl ester was the major compound.

## Introduction

Several studies revealed that secondary metabolites of microbial origin represent unexplored sources for potential bioactive compounds, where endophytes have the ability to synthesize novel lead molecules with diverse structural and abroad bioactivities (Falade et al. 2021; Abdul-Razek et al. 2020; Gunatilaka 2006; Okoye et al. 2015). Plants that contain endophytes are much healthier than endophyte-free plants, since endophytes provide growth and protection for the host plant by different mechanisms (Busby et al. 2016; Rachel et al. 2022). Fungi are plant-like organisms that lack chlorophyll and are represented by several fungal species. There are 1.5 million species approximately, of which 74,000 are known (Hawksworth 2001). Endophytic fungi are present in all kinds of plants, including trees, grasses, algae, and herbaceous plants (Hyde and Soytong 2008, Bacon and White 2000). Several bioactivities of endophytic fungi can be attributed to synthesizing a number of secondary metabolites, including phenolics, alkaloids, quinones, lignans, peptides, flavonoids, steroids, isocoumarins, and flavonoids (Khalil et al. 2021; Talukdar et al. 2021). Many compounds with antimicrobial, antimalarial, and anticancer potential have been isolated from endophytic fungi (Hridoy et al. 2022; Dramae et al. 2022; Li et al. 2018). Therefore, endophytes are a vast repository for an accumulated wealth of unique chemical structures that can be utilized to treat numerous diseases.

The Euphorbiaceae family is one of the largest plant families, with over 330 genera and 8000 species. Several *Euphorbia* plants have been used since ancient times as traditional medicinal herbs in India and China for the treatment of different ailments such as asthma, cough, tumors, and antiviral with diverse chemical classes of secondary metabolites (Diop et al. 2018, Ernst et al. 2015, Olounladé et al. 2017, Zengin et al. 2017). In folk medicine, *Euphorbia milii* has been used to treat hepatitis, warts, and cancer, and it is also reported to have antiviral and cytotoxic properties (Chaman et al. 2019; Delgado et al. 2003) as well as antifungal, antinociceptive, and molluscicidal properties (Opferkuch and Hecker 1982; Rauf et al. 2014). The antiproliferation potential of *E. milii* using an exquisite combination of phytopharmacological and advanced computational techniques revealed that the chloroform fraction of methanol crude extract had the highest antioxidant and significant cytotoxicity (Talha et al. 2020). Phytochemical studies revealed the presence of sterols, sesquiterpenes, triterpenes, phenolic acids, flavonoids, coumarin, alkaloid, and ellagic acid (Hammad et al. 2019; Hemalatha et al. 2015; Marie and Ronnie 2018). The previous studies of endophytic fungi isolated from *Euphorbia* species demonstrated that *Chaetomium strumarium* isolated from *E. hirta* (Anitha and Mythili 2017), *Fusarium* sp. from *E. pekinensis* (Dai et al. 2008), and laccase enzyme producing fungi from *E. milii* (Rao et al. 2018). Endophytic fungi isolated from medicinal plants have not been investigated yet for the discovery of bioactive metabolites, and limited numbers of endophytes from medicinal plants have been reported (Uzma et al. 2019). Therefore, scientists of different disciplines focus on the biological evaluation of fungal secondary metabolites. Only one report investigated the laccase enzyme production ability of endophytic fungi from *E. milli* (Rao et al. 2018). However, antimicrobial and identification of corresponding metabolites have not been previously reported. Plant extracts composition including nine *euphorbia* plants as well as quantification of flavonoids and phenolic acids were previously identified using LC–MS/MS technique (Bouhafsoun et al. 2018; Ceylan et al. 2016; Yener et al. 2018). GC–MS is a powerful tool validation for qualitative and quantitative investigation of some chemical, volatile and thermally stable compounds (Derya et al. 2020; Mehmet et al. 2016).

Therefore, the present study aimed to evaluate the antimicrobial potential of endophytic fungus strains isolated from *E.milii* and identify the main secondary metabolites using GC–MS and LC–MS/MS techniques.

## Materials and methods

### Plant material

The plant sample of *Euphorbia milii* was collected from Orman Garden (30°1ʹ45ʺN, 31°12ʹ47ʺE) Giza, Egypt. Samples were placed in polyethylene bags, labeled, transported to the laboratory, and refrigerated at 4 °C.

### Isolation and purification of endophytic fungi

After surface sterilization of the host plant sample; fresh, healthy parts of leaves and stem of *Euphorbia milii* have been cut into pieces and placed in Potato dextrosw agar (PDA) (Greenfield et al. 2015). After a week of incubation at 28 °C temperature, when fungal mycelium tips were observable, each hyphal tip was transferred onto a fresh PDA slant. For purification of the fungal strains, this step was repeated several times until the colony was deemed uniform. All pure isolates were numbered and stored on the surface of PDA slants at 4 °C (Hanlin 2000). Fungal characterizations were made according to their macroscopic and microscopic characteristics. The cultural and morphological characteristics of the isolate were compared with species descriptions reported in the standard taxonomic manuals to identify the fungal genera and species. Light micrographs were captured using an Olympus CX40 RF100 light microscope coupled to a Canon A620 digital camera (Hanlin 2000). Based on the antagonistic screening; the most active fungal isolate was selected for biological activity studies.

### Genotypic identification of the most active fungal isolates

#### Sequencing and molecular phylogenetics

Based on the preliminary screening; the most potent fungal isolate representing the highest antimicrobial activity was inoculated on PDA and colony morphology was recorded after 48 h of incubation at 25 °C. Microscopic observations were made on the fungus stained with lactophenol cotton blue under the microscope. The molecular characterization was performed through isolation of 18S rDNA. The ITS1–5.8S–ITS2 genomic region was amplified from genomic DNA, using the forward primer ITS1 (5-TCCGTAGGTGAACCTGCGG-3) and the reverse primer ITS4 (5-TCCTCCGCTTATTGATATGC-3) (Hemalatha et al. 2015; White et al. 1990). In short, the reaction conditions for polymerase chain reaction are performed with 2 μl of 10 X Taq PCR buffer, 1.6 μl from 2.5 mM dNTP mixture, 1 μl of both forward and reverse primers (10 pmol/μl), 2 μl template genomic DNA (20 ng/μl), 0.2 μl from KOMATaq. (2.5 U/μl), and distilled water (HPLC grade) up to 20 μl. The reaction mixture was as follows: initial denaturation at 95 °C for 1 min, 30 cycles dent. 95 °C for 30 s, annealing 55 °C for 2 min, extension 68 °C for 1.5 min, final extension 68 °C for 10 min for 1 cycle. After completion, the PCR products were electrophoresed on 1% agarose gels, containing ethidium bromide (10 mg ml), to ensure that a fragment of the correct size had been amplified. The amplification products were purified by Montage PCR clean up kit (Millipore). The purified PCR products were sequenced, using the 2 primers that were used before in the PCR reaction. Sequencing was performed by big Dye Terminator Cycle Sequencing kit V.3.1 (Applied Biosystems, USA). PCR products were resolved on an Applied Biosystems model 3730XL automated DNA sequencing system (Applied Biosystems, USA) at the Macrogen, INC, Seoul Korea.

Phylogenetic data were obtained by aligning the nucleotides of different 18S rRNA retrieved from BLAST algorithm (www.ncbi.nlm.nih.gov/BLAST), MEGA6 BLAST and fast minimum evolution method were used for tree formation. The further parameters in the tree were inserted through MEGA-X software, and the sequence obtained was submitted to NCBI database.

#### Fermentation and crude extract preparation

The most active fungal endophyte was cultivated on PDA medium and incubated at 28 °C, statically. After 21 days of incubation, secondary metabolites were extracted by soaking in methanol 70% overnight. The cultures were filtered under vacuum through filter paper (Whatman No. 4; Brentford, UK). After drying, the extraction process by ethyl acetate was carried out three times for complete extraction. The organic phase was concentrated to dryness under reduced pressure using a rotary evaporator to obtain the crude fungal extract. A part of crude ethyl acetate extract was partitioned using *n*-hexane and the other part was fractionated on column chromatography using silica gel to afforded six sub-fractions (F1–F6). The 6 fractions were obtained by applying ethyl acetate extract on silica gel column using *n*-hexane/ethyl acetate in the order of increasing polarity; 5, 10, 20, 30, 40, and 50%, respectively. TLC was used to collect the similar fractions and detection was performed under UV lamp (254 and 356 nm) as well as spraying reagents. TLC showed a major compound for F2 which further purified on Sephadex LH-20 using chloroform/methanol followed by two successive column on silica gel using dichloromethane/methanol (8:2) to afford a crude compound 1 (1.5 mg).

#### In vitro antibacterial and antifungal assay

Antimicrobial activity was measured by an agar diffusion method against Gram-positive bacteria (*Bacillus subtilis* ATCC 6633), Gram-negative bacteria (*Escherichia coli* ATCC 25,922), unicellular yeast fungi (*Candida albicans* ATCC 10,321), and filamentous fungi (*F. solani* NRC15 and *F. oxysporum*). Bacteria and yeast strains were obtained from the American Type Culture Collection, while fungi were obtained from the culture collection of the Chemistry of Natural and Microbial Products Department, National Research Centre, Egypt. Thiophenicol (Thiamphenicol, Sanofiaventis, France) and Treflucan (Fluconazole, Egyptian Pharmaceutical Industries Company EIPICO) were used as antibacterial and antifungal positive controls, respectively, in a concentration of 100 μg/disk and DMSO was used as a negative control. The disks (1 mg/5 μl) were applied on inoculated nutrient agar and PDA media plates and incubated for 24 h at 37 °C for bacteria and 72 h at 28 °C for fungal strains, respectively. The zone of inhibition was recorded and compared with the positive control treatments.

#### MIC of endophytic strains and their fractions

Endophytic strain *C. ovatoascomatis NRC*, *n*-hexane fraction, and sub-fractions F1-F6 were screened in vitro against Gram-positive bacteria (*Bacillus subtilis* ATCC 6633, *Staphylococcus aureus* ATCC29213 and *Lactobacillus cereus ATCC14579)*, Gram-negative bacteria (*Escherichia coli* ATCC 25922 and *Salmonella enteric* ATCC 25566), unicellular yeast fungi (*Candida albicans* ATCC 10321), and filamentous fungi (*Aspergillus niger* NRC53, *F. solani* NRC15 and *F. oxysporum*). The minimum inhibitory concentration of each sample against tested microorganisms was determined by microtiter broth dilution method: MIC was performed on the extracts, fractions and isolated compounds by serial dilution as previously described (Eloff 1998; Teh et al. 2017) according to the Clinical and Laboratory Standards Institute (CLSI) guidelines. The serial dilutions from the stock solution were made ranges 500, 250, 125 and 62.5 using Nutrient broth and Potato dextrose broth for Bacteria and fungi, respectively, in 96-well microplates. The bacterial suspension containing approximately 5 × 10^5^ colony-forming units/mL was prepared from a 24 h culture plate. From this suspension, 100 μl was inoculated into each well. A sterility control well and a growth control well were also studied for each strain. Thiophenicol and Treflucan were used as positive controls for antibacterial and antifungal activity in a concentration of 500, 250, 125 and 62.5 μg. The microtiter plates were incubated at 37 °C, 24 h for bacteria and 28 °C, 48 h for fungi. After incubation; the MIC values visually determined. The lowest concentration of each extract displaying no visible growth was recorded as the minimum inhibitory concentration.

#### GC–MS analysis

GC–MS analysis was performed using a Thermo Scientific, Trace GC Ultra/ISQ Single Quadrupole MS, TG-5MS fused silica capillary column (30 m, 0.251 mm, 0.1 mm film thickness). For GC–MS detection, an electron ionization system with ionization energy of 70 eV was used, Helium gas was used as the carrier gas at a constant flow rate of 1 mL/min. The injector and MS transfer line temperature was set at 280 °C. The oven temperature was programmed at an initial temperature 50 °C (hold 2 min) to150 °C at an increasing rate of 7 °C/min then to 270 at an increasing rate 5 °C/min (hold 2 min) then to 310 as a final temperature at an increasing rate of 3.5 °C/min (hold 10 min). The quantification of all the identified components was investigated using a percent relative peak area. A tentative identification of the compounds was performed based on the comparison of their relative retention time and mass spectra with those of the NIST (National Institute of Standards and Technology, Gaithersburg, MD, USA), WILEY library data of the GC–MS system.

### LC-ESI-TOF–MS analysis

#### Sample preparation

A stock solution of the extract was prepared from 50 mg of the lyophilized ethyl acetate extract dissolved in 1000 µL of the solvent mixture composed of water: methanol: acetonitrile (H_2_O:MeOH: ACN) in a ratio of 2:1:1. Complete solubility of stock solution was obtained by sample vortexed and ultra-sonication at 30 kHz for 10 min. An aliquot, 20 µL of the stock solution was again diluted with 1000 µL of the H_2_O: MeOH: ACN (2:1:1) and centrifuged at 10,000 rpm for 5 min, and 10 µL (1 µg/mL) was used for injection. The LCMS analysis was also performed for blank and quality control samples/internal standard (IS) for confidence in the experiment. The sample was injected in negative mode.

#### Instruments and acquisition method

Small molecules were separated on an ExionLC system (AB Sciex, Framingham, MA, USA) connected with an autosampler system, an in-line filter disks pre-column (0.5 µm × 3.0 mm, Phenomenex, Torrance, CA, USA), and an Xbridge C18 (3.5 µm, 2.1 × 50 mm) column (Waters Corporation, Milford, MA, USA) was maintained at 40 °C, and at a flow rate of 300 µL/min was utilized. The mobile phase consisted of solution (A): 5 mM ammonium formate in 1% methanol, pH adjusted to 8.0 by using sodium hydroxide, and solution (B): consisting of acetonitrile (100%). The gradient elution was performed with the following program: 0–20 min, 10% B; 21–25 min, 90% B; 25.01–28 min, 10% B; and then 90% B for equilibration of the column. The mass spectrometry (MS) was performed on a Triple TOF 5600 + system equipped with a Duo-Spray source operating in the ESI mode (AB SCIEX, Concord, ON, Canada). The sprayer capillary and declustering potential voltages were − 4500 and − 80 V. The source temperature was set at 600 °C, the curtain gas was 25 psi, and gas 1 and gas 2 were 40 psi. The collision energy − 35 V (negative mode) with CE spreading 20 V and the ion tolerance for 10 ppm were used. The TripleTOF5600 + was operated using an information dependent acquisition (IDA) protocol. Batches for MS and MS/MS data collection were created using Analyst-TF 1.7.1. The IDA method was used to collect full-scan MS and MS/MS information simultaneously. The method consisted of high-resolution survey spectra from 50 to 1100 m/z and the mass spectrometer was operated in a pattern where a 50-ms survey scan was detected. Subsequently, the top 15 intense ions were selected for acquiring MS/MS fragmentation spectra after each scan (Fayek et al. 2019).

#### LC–MS data processing

MS-DIAL 3.70 open-source software (Tsugawa et al. 2015) was used for non-targeting, small molecule comprehensive analysis of the sample. According to the acquisition mode, ReSpect negative (1573 records) databases were used as reference databases. The search parameters were set as MS1 and MS2 mass tolerance: 0.01 Da and 0.05 Da for data collection, for peak detection; minimum peak height: 100 amplitude, mass slice width: 0.05 Da, smoothing level: 2 scans, minimum peak width: 6 scans, for identification MS1 and MS2 tolerance: 0.2 Da/each, for alignment; retention time tolerance: 0.05 min and MS1 tolerance: 0.25 Da. The MS-DIAL output was used to run again on PeakView 2.2 with the MasterView 1.1 package (AB SCIEX) for feature (peaks) confirmation from Total Ion Chromatogram (TIC) based on the criteria; aligned features having signal-to-noise ratio greater than 5 and intensities of the sample: blank greater than 5.

## Results and discussion

### Microorganisms isolation and antimicrobial screening

*Euphorbia milii* yielded a total of 5 fungal isolates belonging to the following genera: *Alternaria, Aspergillus, Fusarium,* and *Chaetomium*. Antimicrobial assays of microorganism’s fungal isolates are shown in Table 1. The ethyl acetate extract of fungal isolate coded (MP2) *Chaetomium ovatoascomatis* revealed a promising activity against Gram-negative *E. coli* ATCC25922, and Gram-positive *B. subtilis* with inhibition zones 15 and 22 mm, respectively, and showed moderate activity against tested yeast *C. tropicalis* with inhibition zone 7 mm as well as a considerable effect against tested fungal strains *F. solani* and* F. oxsporium* with inhibition zone 7 and 13 mm, respectively. The strain *Chaetomium ovatoascomatis* was selected for phytochemical investigation using LC–MS/MS and GC–MS analysis based on the screening process results.

**Table 1:** Antimicrobial assays of microorganism's fungal isolates

Pathogenic	Inhibition zone diameter (mm)				
	G + bacteria	G − bacteria	Yeast	Fungi	
Isolate Code	*B. subtilis*	*E. coli*	*C.tropicalis*	*F. solani*	*F. oxsporium*
MP1^a^	7	15	7	8	7
MP1(WB^b^)	7	19	6	8	10
MP2^a^	22	15	7	8	13
MP2 (WB^b^)	21	12	7	22	7
MP3*	9	10	N. A	13	7
MP3 (WB^b^)	7	13	N. A	8	6
PP1^a^	6	6	8	15	6
PP1 (WB^b^)	7	15	7	8	6
PP2^a^	7	12	7	7	6
PP2 (WB^b^)	7	13	6	N. A	7
PC	29	10	14	15	11

The inhibition zone diameter expressed in (mm). Thiophenicol and Treflucan were used as positive controls at a concentration of 100 μg/disk, DMSO was used as negative control

^a^Refers to that the fungal extract from fungi cultivated on PDA

^b^Refers to that the fungal extract from fungi cultivated on Media Contain wheat bran as carbon source

### Identification of the most active isolates based on molecular phylogenetic analysis

**Fig. 1:**
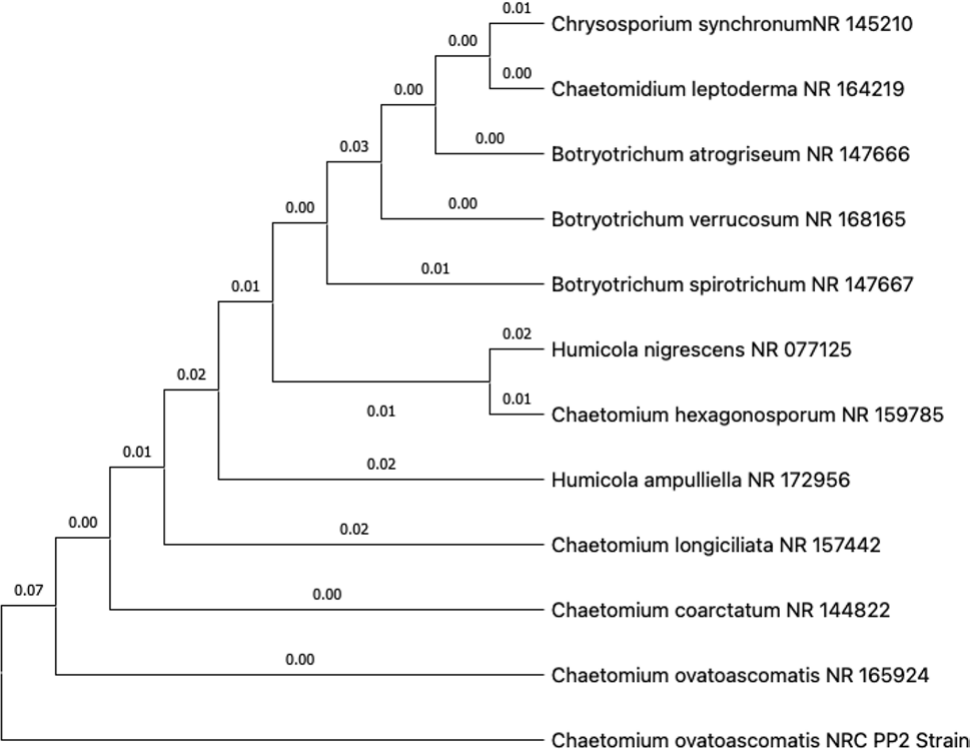
Phylogenetic analysis of fungal strains

Morphological identification revealed the formation of colonies on PDA fast-growing, reaching around 7 cm diam. After seven days after incubation at 25 °C, the colony was sparse, rough, raised with an entire edge, thinly hairy, and well defined at the margin; colony from above; rough, sparse, whitish; from below, pale brown. Sporulate on PDA, these morphological characteristics agreed with (Raza et al. 2019). The phylogenetic analysis of the isolated fungal strain MP2 *Chaetomium ovatoascomatis* revealed 98% identity with ITS sequences of rRNA genes of related species using BLAST programs (Fig. 1). (Selim et al. 2014).

### Phytochemical analysis

Endophytic fungi are an abundant source of secondary metabolites such as coumarins, terpenoids, glycosides, and flavonoids, which contain a multitude of antifungal, anticancer, and antibacterial compounds. Terpenoids demonstrated several pharmacological effects, including antibacterial, antitumor, antiviral, anti-inflammatory as well as cardiovascular disease treatment (Yang et al. 2020). Flavonoids possess antifungal activity via diverse mechanisms such as mitochondria and plasma membrane destruction (Al Aboody and Mickymaray 2020). Flavonoids exert their antibacterial effect by damaging the cytoplasmic membrane, thereby impeding the synthesis of nucleic acids (Zhenyou et al. 2022). Phytochemical analysis of ethyl acetate extract of *Chaetomium ovatoascomatis NRC* and its nonpolar hexane fraction was performed by LC–MS/MS and GC–MS, respectively.

### Identification of the crude compound 1 of Chaetomium ovatoascomatis NRC

**Fig. 2:**
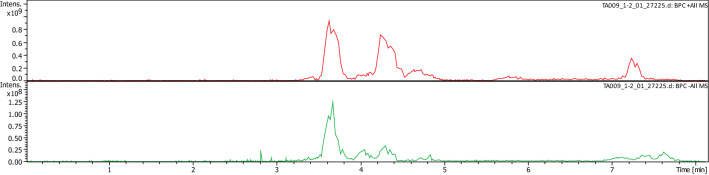
LC–MS of a crude compound 1 in positive (above) and negative mode

**Fig.3:**
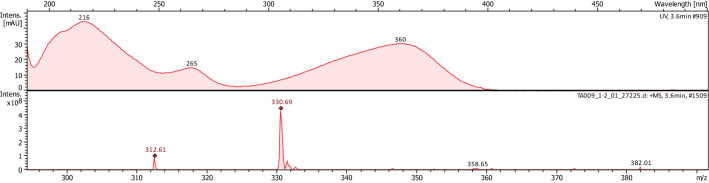
UV absorbance (above) and mass spectrum for crude compound 1 in positive mode

**Fig. 4:**
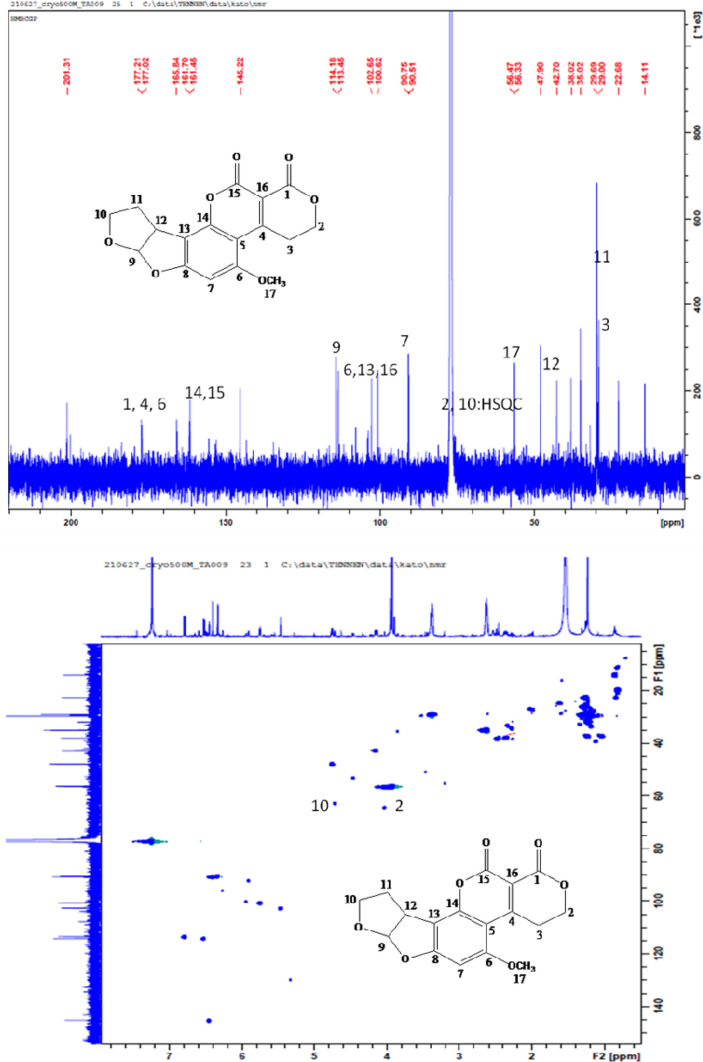
^13^C–NMR (above) and HSQC spectrum of crude compound 1

The major crude compound **1** was identified by a combination of LC–MS, LC–MS/MS, and NMR tools. Negative and positive mode LC–MS analysis of crude **1** showed molecular ion peak at m/z 328 and 330, respectively, suggesting molecular weight of 329 Daltons (Fig. 2, 3). LC–MS/MS analysis (negative mode) of *Chaetomium ovatoascomatis* total extract was used to predict the molecular formula corresponding to m/z 329 by using PeakView software program which suggested molecular formula of C_17_H_13_O_7_, [M–H]^−^. The precursor ion peak m/z 329.066 exhibited to daughter ions at m/z 314, 311, 301, 283 corresponding to [M–H–CH3]^–^, [M–H–H_2_O]^–^, [M–H–CO]^–^, [M-H–CO–H_2_O]^–^, respectively. Analysis and comparison to the references public database HMBD and FOODB for precursor and daughter ions peaks were consistent with aflatoxin G2. ^13^C–NMR and HSQC of crude compound **1** exhibited the unresolved mixture with expected signals of aflatoxin G2 (Fig. 4).

### LC–MS/MS of ethyl acetate extract of Chaetomium ovatoascomatis NRC

**Fig. 5:**
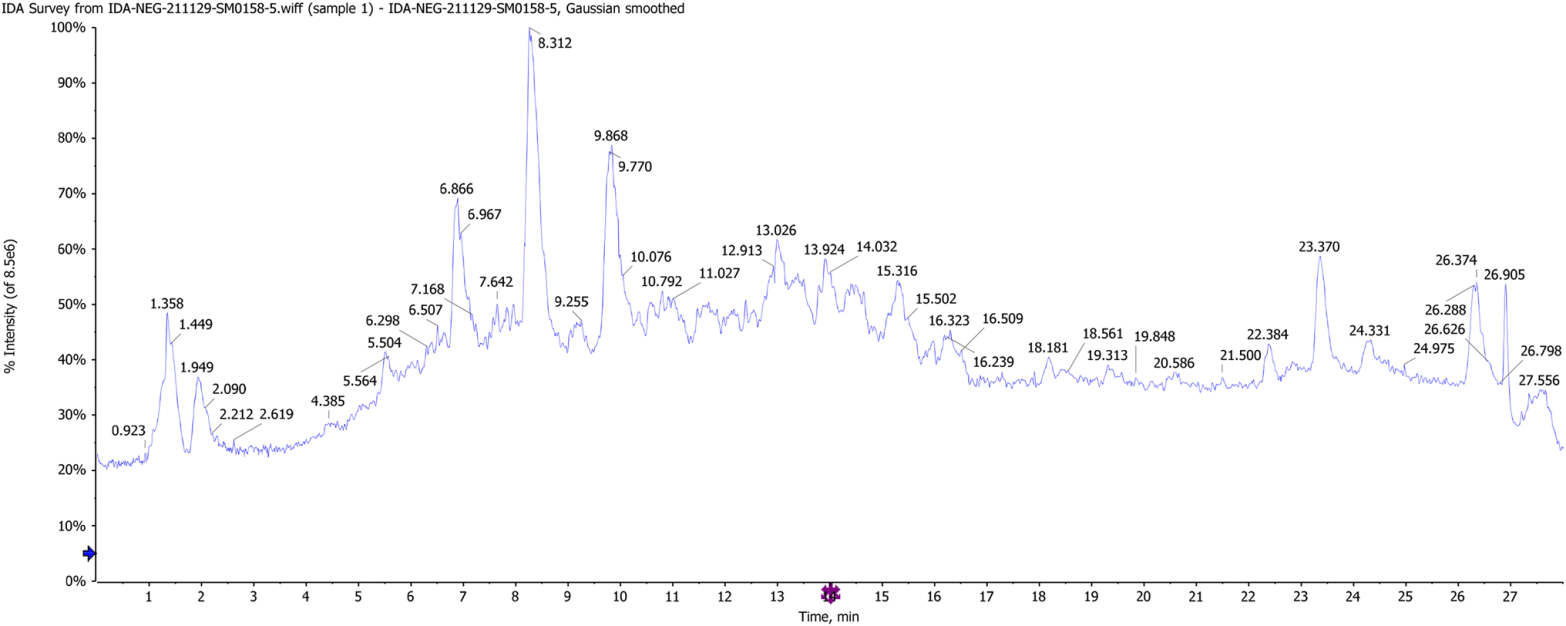
Chromatogram of LC–MS/MS of ethyl acetate extract of *Chaetomium ovaoascomatis*

LC–MS/MS analysis of ethyl acetate extract (Fig. 5) revealed the presence of an additional compound with precursor ion m/z 339.0499 with a predicted molecular formula of C_18_H_11_O_7_ [M–H]^–^. The daughter ions were shown at m/z 321 (C_18_H_9_O_6_), 307 (C_17_H_7_O_6_), 295 (C_17_H_11_O_5_), 265 (C_16_H_9_O_4_). Comparison to the references public database (HMBD, FOODB) for the precursor ion and fragment daughter ions suggested the presence of grevilline B which belongs to catechols.

**Fig.6:**
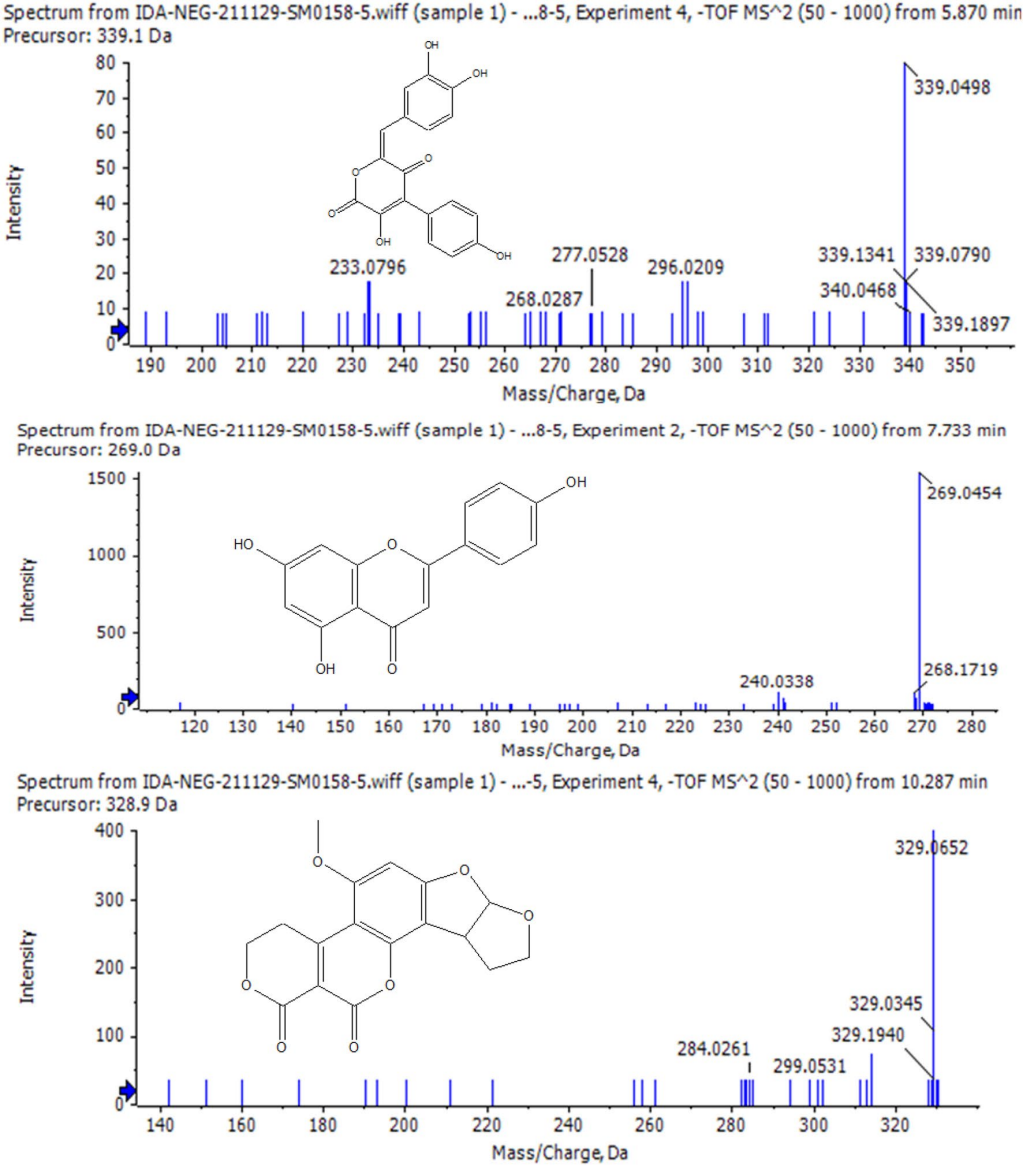
Mass spectrum of grevilline B, apigenin and aflatoxin G2

The same procedures were followed, and a number of metabolites were tentatively identified (Table 2) by comparison with the public reference database and reported data, including austdiol, apigenin, citreaoisocoumarin, macrosporin, quinalizatrine, alternariol, and questinol. The mass spectrum of the major identified compounds is shown in Fig. 6.

**Table 2:** LC–MS/MS of *Chaetomium ovatoascomatis NRC*, Adduct ion as [M–H]^–^

	RT	Precursor ion m/z	Error PPM	Formula	MS/MS	Identified compound	
1	5.87	339.049	− 0.3	C_18_H_11_O_7_	321,307,295,265	Grevilline B	Allen et al. (2015)
2	7.52	235.060	2.5	C_12_H_11_O_5_	207,191,189,146.9	Austdiol	Vishwanath et al. (2009)
3	7.73	269.045	3.4	C_15_H_9_O_5_	251,242,225,151,117	Apigenin	Razgonova et al (2021),
4	7.97	277.072	2.5	C_14_H_13_O_6_	220,219,191	Citreaoisocoumarin	Hassan (2007)
5	9.1	283.061	4.3	C_16_H_11_O_5_	268,240,211	Macrosporine	Varga et al. (2013)
6	10.1	271.024	1.7	C_14_H_7_O_6_	253,243,229	Quinalizatrine	Allen et al. (2015)
7	10.29	329.065	− 1.2	C_17_H_13_O_7_	311,229,283	Aflatoxin G2	Tsiplakou et al. (2014)
8	10.34	299.055	0.7	C_16_H_11_O_6_	284,281,267,255, 237	Questinol	Allen et al. (2015)
9	11.11	257.046	7	C_14_H_9_O_5_	241,215,213,189	Alternariol	Varga et al. (2013)

### GC–MS analysis

GC–MS analysis of the nonpolar hexane fraction tentatively identified 30 compounds (Table 3), where 9,12-octadecadienoic acid methyl ester was the major compound (35.6%), followed by 9-octadecenoic acid methyl ester (16.1%) then hexadecanoicacid methyl ester (13.9%). In the range of 4.94 to 3.10 percent, additional compounds were tentatively identified as 2-ethyl4-methyl-2, 5-dihydrooxazole, l-Limonene, 2-Methyl-2(1,4-dimethylcyclohexyl) cyclopentanone, and octadecanoic acid, methyl ester.

**Table 3:** GC–MS of *n*-hexane fraction

	RT	Probability	Compound Name	Area %	Mol. Wt	Formula
1	5.18	4.61	2-ethyl4-methyl-2,5-dihydrooxazole	4.94	113	C_6_H_11_NO
2	5.82	90.07	2,5-Dibromo1,4-dinhexadecylbenzene	0.11	682	C_39_H_69_Br_2_
3	9.18	22.95	l-Limonene	4.39	136	C_10_H_16_
4	22.35	40.39	Phenol,2,4-bis(1,1dimethylethyl)	0.30	206	C_14_H_22_O
5	24.40	12.38	3-Butenamide	0.16	85	C_4_H_7_NO
6	27.40	47.00	2-Methyl-2(1,4-dimethylcyclohexyl)cyclopentanone	3.98	208	C_14_H_24_O
7	28.19	11.06	1-Heptadecene	0.16	238	C_17_H_34_
8	28.66	9.86	2-Pyridinecarboxylic acid, 2-acetylhydrazide	0.14	179	C_9_H_9_N_3_O_2_
9	28.94	16.06	Pentadecanoicacid methyl ester	0.28	256	C_16_H_32_O_2_
10	29.89	16.64	1,2-Benzenedicarboxylic acid, bis(2methylpropyl)ester	0.26	278	C_16_H_22_O_4_
11	30.46	8.68	10-Undecenoic acid methyl ester	0.16	198	C_12_H_22_O_2_
12	30.91	41.85	Hexadecanoicacid methyl ester	13.90	270	C_17_H_34_O_2_
13	31.73	5.07	1,2-Benzenedicarboxylic acid, monobutyl ester	0.51	222	C_12_H_14_O_4_
14	32.09	21.28	4-oxo, 2-hexenal-7	0.50	112	C_6_H_9_O_2_
15	32.19	21.66	Pentadecanoic acid, 4,6,10,14tetramethyl, methyl ester	0.21	312	C_20_H_40_O_2_
16	32.35	8.39	9-Dodecenoic acid, methyl ester,	0.18	212	C_13_H_24_O_2_
17	32.79	36.35	Heptadecanoic acid methyl ester	0.90	284	C_19_H_36_O_2_
18	33.63	3.36	17-Pentatriacontene	0.13	490	C_35_H_7_
19	33.87	9.74	1-Heptadecanol	0.18	256	C_17_H_36_O
20	34.08	31.32	9,12-Octadecadienoic acid (Z,Z) methyl ester	35.62	294	C_19_H_34_O_2_
21	34.16	10.84	9-Octadecenoic acid (Z),methyl ester	16.09	296	C_19_H_36_O_2_
22	34.59	29.00	Octadecanoic acid, methyl ester	3.10	298	C_19_H_39_O_2_
23	35.29	95.12	Cyclohexane 1,3,5-trimethyl-2-octadecy	0.12	378	C_27_H_54_
24	40.16	93.16	Tetratertbutyl 2,6-di(3-propenyl) 3,7dimethoxybicyclo[3.3.0]octa3,7diene2,4,6,8-dicarboxylate	0.13	646	C_36_H_54_O_10_
25	40.56	4.70	1-Hexanol,2-ethyl	0.22	130	C_8_H_18_O
26	41.49	31.23	Di(2-ethylhexyl)phthalate	9.70	390	C_24_H_38_O_4_
27	42.05	4.55	Undecane	0.11	156	C_11_H_24_
28	43.49	5.25	Docosane	0.12	310	C_22_H_46_
29	47.02	5.71	Octanal	0.15	128	C_8_H_16_O
30	47.30	3.43	1-Decanol,2-hexyl	0.14	242	C_16_H_34_O

### Antibacterial activity

Endophyte fungi are promising sources of novel bioactive compounds that can be utilized for pharmaceutical, medicinal and agricultural applications (Manganyi and Ateba 2020)**.** Several studies on fungal endophytes have reported significant antimicrobial activity due to the presence of different classes of bioactive molecules (Rao et al. 2015; Rodriguez et al. 2009). In the present study, ethyl acetate extract of *Chaetomium ovatoascomatis NRC*, *n*-hexane fraction, and sub-fractions (1F-6F) was evaluated against Gram-positive bacteria (*Bacillus subtilis* ATCC 6633, *Staphylococcus aureus* ATCC29213 and *Lactobacillus cereus* ATCC14579), Gram-negative bacteria (*Escherichia coli* ATCC 25922 and *Salmonella enteric* ATCC 25566), unicellular yeast fungi (*Candida albicans* ATCC 10321), and filamentous fungi (*Aspergillus niger* NRC53, *F. solani* NRC15 and *F. oxysporum*). Antibacterial results (Table 4) revealed a promising activity of ethyl acetate extract of *Chaetomium ovatoascomatis* against Gram-negative (*E. coli* ATCC25922, *Salmonella enteric* ATCC 25,566) and *Aspergillus niger* NRC53 with MIC of 62.5ug and considerable effect against Gram-positive (*Staphylococcus aureus* ATCC29213,* Lactobacillus cereus* ATCC14579) and *Candida albicans* ATCC 10321 with MIC value of 125.0ug. The most significant activity was recorded for *n*-hexane and F2 fractions against Gram-positive, Gram-negative, yeast, and filamentous fungi with MIC of 62.5 ug for both fractions. Whereas, *n*-hexane has higher activity against *F. solani* NRC15 while F2 is more effective against *Aspergillus niger* NRC53. F4 fraction demonstrated its activity against 2 Gram-positive (*Bacillus subtilis* ATCC 6633, *Lactobacillus cereus* ATCC14579.) and two Gram-negatives (*Escherichia coli* ATCC 25922, *Salmonella enteric* ATCC 25566) and *Aspergillus niger* NRC53. F1 exhibited antibacterial activity against Gram-positive (*Staphylococcus aureus* ATCC29213, *Bacillus subtilis* ATCC 6633) and Gram-negative (*Escherichia coli* ATCC 25922, *Salmonella enteric* ATCC 25566). The antimicrobial activity of fractions F3, F5, and F6 was significantly lower than that of the previous fractions. The antimicrobial activity of ethyl acetate extract of *Chaetomium ovatoascomatis* is due to the presence of major metabolites belonging to aflatoxin, catechol, and flavonoid classes. Aflatoxin G2 is a highly toxic secondary metabolite previously produced by *Chaetomium* species (Koyama et al. 1991). Grevilline B belongs to catechols compounds demonstrating significant antimicrobial activity (Jeong et al. 2009). Apigenin has several medicinal properties, including antiviral, antibacterial, and antioxidant (Thirukumaran et al. 2019), as well as significant antimicrobial activities of flavonoids (Zhenyou et al. 2022; Al Aboody and Mickymaray 2020). The antimicrobial results of *n*-hexane can be attributed to the presence of 9,12-octadecadienoic acid methyl ester and 9-octadecenoic acid methyl as major constituents consistent with the previously reported activity of fatty acid methyl esters (Chandrasekaran et al. 2008; Agoramoorthy et al. 2007). Currently, chromatographic identification of the obtained fractions is being conducted to determine the chemical constituents and rationalize their antimicrobial activities.

**Table 4:** MIC of fungal endophyte strain and their fractions

	G + ve			G - ve		Yeast	Fungi		
	*Staphylococcus aureus* ATCC29213	*Bacillus subtilis* ATCC 6633	.*Lactobacillus cereus *ATCC14579	*Escherichia coli* ATCC 25922	*Salmonella enteric* ATCC 25566	*Candida albicans* ATCC 10321	*Aspergillus niger* NRC53	*F. solani* NRC15	*F. oxysporum*
*C.ovatoascomatis* Ethyl acetate	125	250	125	62.5	62.5	125	62.5	250	250
*n*-hexane	250	62.5	62.5	62.5	62.5	62.5	125	62.5	62.5
F1	62.5	62.5	125	62.5	62.5	125	500	125	125
F2	250	62.5	62.5	62.5	62.5	62.5	62.5	125	62.5
F3	125	*NA	*NA	125	*NA	250	125	*NA	*NA
F4	250	62.5	62.5	62.5	62.5	250	62.5	125	125
F5	250	62.5	*NA	62.5	*NA	500	62.5	*NA	*NA
F6	125	*NA	*NA	62.5	*NA	250	250	*NA	*NA
**PC	125	125	125	125	125	125	125	125	125

*NA Not Detected, **PC: Positive Control

## Conclusion

*Chaetomium ovatoascomatis NRC* strain was isolated herein for the first time from *E.milli* and demonstrated promising antimicrobial activity against tested pathogens. In addition, *n*-hexane and sub-fractions of the strain exhibited broader activity against Gram-positive and Gram-negative bacteria, yeast, and filamentous fungi with a higher MIC value than the positive control. LC–MS/MS of ethyl acetate extract of *Chaetomium ovatoascomatis* revealed the presence of aflatoxin G2, grevilline B, and apigenin with potential antimicrobial activity. The GC–MS analysis of *n*-hexane revealed 9,12-octadecadienoic acid methyl ester as the major antimicrobial component. The combination of LC–MS/MS and NMR would be helpful in identifying impure metabolites with few milligrams.

## Data Availability

The datasets generated during and/or analyzed during the current study are available from the corresponding author on reasonable request.

## Abbreviations

E. milii: Euphorbia milii
GC–MS: Gas Chromatography/Mass spectrometry
LC–MS/MS: Liquid Chromatography with tandem mass spectrometry
MIC: Minimum Inhibition Concentration
NMR: Nuclear magnetic Resonance
^1^H: Proton magnetic resonance
^13^C: Carbon magnetic resonance
HSQC: Heteronuclear Single-Quantum Correlation Spectroscopy (J_2_^1^H-^13^C-correlations)
NRC: National Research Centre
